# Secure and Efficient Regression Analysis Using a Hybrid Cryptographic Framework: Development and Evaluation

**DOI:** 10.2196/medinform.8286

**Published:** 2018-03-05

**Authors:** Md Nazmus Sadat, Xiaoqian Jiang, Md Momin Al Aziz, Shuang Wang, Noman Mohammed

**Affiliations:** ^1^ Department of Computer Science University of Manitoba Winnipeg, MB Canada; ^2^ Department of Biomedical Informatics University of California San Diego La Jolla, CA United States

**Keywords:** privacy-preserving regression analysis, Intel SGX, somewhat homomorphic encryption

## Abstract

**Background:**

Machine learning is an effective data-driven tool that is being widely used to extract valuable patterns and insights from data. Specifically, predictive machine learning models are very important in health care for clinical data analysis. The machine learning algorithms that generate predictive models often require pooling data from different sources to discover statistical patterns or correlations among different attributes of the input data. The primary challenge is to fulfill one major objective: preserving the privacy of individuals while discovering knowledge from data.

**Objective:**

Our objective was to develop a hybrid cryptographic framework for performing regression analysis over distributed data in a secure and efficient way.

**Methods:**

Existing secure computation schemes are not suitable for processing the large-scale data that are used in cutting-edge machine learning applications. We designed, developed, and evaluated a hybrid cryptographic framework, which can securely perform regression analysis, a fundamental machine learning algorithm using somewhat homomorphic encryption and a newly introduced secure hardware component of Intel Software Guard Extensions (Intel SGX) to ensure both privacy and efficiency at the same time.

**Results:**

Experimental results demonstrate that our proposed method provides a better trade-off in terms of security and efficiency than solely secure hardware-based methods. Besides, there is no approximation error. Computed model parameters are exactly similar to plaintext results.

**Conclusions:**

To the best of our knowledge, this kind of secure computation model using a hybrid cryptographic framework, which leverages both somewhat homomorphic encryption and Intel SGX, is not proposed or evaluated to this date. Our proposed framework ensures data security and computational efficiency at the same time.

## Introduction

Machine learning algorithms are now being widely used in many applications to uncover deep and predictive insights from datasets that are large scale and diverse. For instance, building predictive models from biomedical data is very important in biomedical science. Such predictive models can identify genetic risk factors for a specific disease under study and can guide medical treatment. For instance, Tabaei and Hermana formulated a predictive equation to screen for diabetes [1].

Machine learning thrives on growing datasets. In most of the cases, the more data fed into a machine learning system, the more it can learn and offer the potential to make more accurate prediction. It is often known as “data never hurt in machine learning,” as insufficient information cannot lead to powerful learning systems. In the context of health care, building an accurate predictive model depends on the quality and quantity of aggregate clinical data, which come from different hospitals or health care institutions. Consequently, in a real-world scenario, machine learning applications use data from several sources, including genetic and genomic, clinical, and sensor data. Day by day, many new sources of data are becoming available—for instance, data from cell phones [2], wearable sensors [3], and participatory sensing applications [4]. For instance, there are wearable sensing frameworks that collect sensing information regarding heart rate, body temperature, caloric expenditure, etc, to train machine learning models. These models are then used for predictive analysis [4].

Data collection, storage, and processing power of a single institution is not always adequate to handle the large-scale data used in cutting-edge machine learning applications. For rare diseases, individual institutions oftentimes do not have sufficient data to calculate a model to achieve sufficient statistical power. Therefore, data sharing among multiple institutions is required. However, sharing sensitive biomedical data (clinical or genomic) exposes many security and privacy threats [5]. In case of data breach, there is a risk of sensitive personal information leakage. Therefore, in addition to addressing the fundamental goal of information retrieval, privacy-preserving learning also requires the learning algorithm to protect the confidentiality of the sensitive records of individuals. Along with obtaining the approval from an institutional review board, collaborative research on shared biomedical data often needs to satisfy 2 criteria at the same time: (1) permitting access to biomedical data for collaborative research, and (2) maintaining participants’ privacy and protecting the confidentiality of their genomic and clinical profile [6]. For this reason, strict policies regarding biomedical data sharing have been enforced and, generally, these policies are different in different regions of the world. For instance, there are several key differences between the US Health Insurance Portability and Accountability Act (HIPAA) and the Canadian Personal Information Protection and Electronic Documents Act (PIPEDA). This difference in the policies and regulations of cross-border biomedical data sharing impedes international research projects greatly [7]. It is imperative to address this problem with practical solutions to promote health science discoveries.

In this paper, we concentrate on secure and efficient computation for a fundamental technique used in numerous learning algorithms called *regression* (see Methods). Regression analysis identifies the correlation among different attributes based on input data. Given a number of high-dimensional data points, regression analysis generates a best-fit line or curve through these points. To evaluate the fit, the value of a target attribute is predicted, which is associated with the given values of input. For instance, the input variables can be an individual’s age, weight, sex, body mass index, and glucose level, while the output can be the likelihood to develop diabetes. Although regression analysis is widely used in practice, little work has been done in privacy-preserving regression analysis over a distributed dataset. Our objective was to perform the required computation for regression analysis without exposing any other information of user data.

### Prior Works

To ensure the security and privacy of the sensitive data used in learning algorithm, different techniques (eg, garbled circuit [8], homomorphic encryption [9], differential privacy [10], and secure hardware [11]) have been adopted (Multimedia Appendix 1 discusses prior works targeting regression). But each of these techniques has certain shortcomings (eg, computational overhead, communication overhead, storage overhead, reduced data utility, and approximation error), which make these techniques difficult to use in real-world applications.

Wu et al developed a framework, grid binary logistic regression (GLORE) [12], for developing a binary logistic regression model where data are distributed across different data owners. In their proposed approach, instead of sharing patient records, data owners send intermediary results to a central entity. These intermediary results are then used to build a prediction model without sharing patient-level data. However, in their approach, the intermediary results are exchanged in plaintext. If the data size of a data owner is small, then sharing the intermediary results might compromise privacy.

Later, Shi et al incorporated secure multiparty computation in GLORE. Their proposed framework, secure multiparty computation framework for grid logistic regression (SMAC-GLORE) [13], protects the confidentiality of intermediary results beside the patient data. However, SMAC-GLORE cannot handle numbers outside of a predefined range, and it does not scale well (eg, it cannot efficiently handle data with more than 10 covariates). In addition, it uses a Taylor series approximation approach to evaluate the logit function. This approximation causes precision loss in the final output.

### Why Hybrid?

There are two obvious but suboptimal solutions in terms of security and efficiency. Existing fully homomorphic encryption (FHE) techniques [14] provide rigorous security, but these solutions are not efficient. In existing homomorphic encryption schemes, with subsequent homomorphic operations, the noise (and size) of the ciphertext grows substantially, which increases computational and storage overheads to a great extent (see Methods, Homomorphic Encryption for details). There are some operations to reduce the size and noise of the ciphertext: *bootstrapping* [9] and *relinearization* [15]. However, these operations are very expensive from the computational point of view. Our proposed framework does not use these expensive operations at all, which enhances the efficiency of the framework greatly.

On the contrary, Software Guard Extensions (SGX; Intel)-based solutions are very efficient but have some security concerns resulting from the recent discovery of side-channel attacks against SGX [16]. We developed our method so that only intermediary results, not individual records, are decrypted inside the secure hardware. Hence, a successful adversary would be unable to compromise the privacy of an individual.

Our proposed hybrid framework uses both techniques and provides a good trade-off in terms of security and efficiency.

### Contributions

In this paper, we propose a hybrid cryptographic framework for secure and efficient regression analysis (both linear and logistic). Our proposed framework leverages the best features of two secure computation schemes: somewhat homomorphic encryption (SWHE) and secure hardware (Intel SGX). In this framework, data reside at the data owner’s end. We assumed that data are horizontally partitioned, where all the records share same attributes. Inspired by GLORE [12], we formulated the regression problem as decomposable parts. Data owners compute these decomposable intermediary results locally. Then, after encrypting these local results using homomorphic encryption, they send the encrypted intermediary results to an SGX-enabled central server. The central server now combines the intermediary results using a homomorphic addition operation. Then, these aggregate encrypted intermediary results are passed to the secure hardware hosted at the central server. Here, the aggregate intermediary results are decrypted and further computation is performed on plaintext. These computations involve matrix inversion and division, which are hard to handle in existing homomorphic encryption schemes. Finally, model coefficients are computed inside the secure hardware.

We summarize our contributions as follows: (1) We address the limitations of existing secure computation schemes and propose a hybrid secure computation model for performing regression analysis over distributed data, which is more efficient and robust. (2) We designed the framework in such a way that no homomorphic multiplication is necessary, which is an expensive operation. In addition, we do not need any bootstrapping or relinearization operation. (3) In our proposed approach, a significant portion of computation is performed at the data owner’s end on plaintext. In computation at a central server, after homomorphic addition operations, further computation is performed inside secure hardware on plaintext. Since most of the operations are performed on plaintext, our proposed approach is very efficient. In addition, due to avoiding any kind of approximation technique, our proposed method does not introduce any precision loss in the final output.

In Multimedia Appendix 1 we introduce major existing secure computation techniques, application of these techniques in regression analysis, and their shortcomings.

## Methods

### Security Background

#### Homomorphic Encryption

The idea of an encryption scheme that is capable of performing arbitrary computation on encrypted data was first proposed by Rivest et al [17] in 1978. Since then, several cryptosystems were invented that are homomorphic with respect to either addition or multiplication. Finally, Boneh et al [18] proposed a partially homomorphic cryptosystem that is able to perform 1 multiplication and any number of additions. Table 1 shows a partial list of homomorphic encryption schemes [18-22].

Developing an encryption scheme that supports an arbitrary number of additions and multiplications was an open problem until 2009. Since addition and multiplication operations over integer ring *Z*_2_ form a complete set of operations, this type of encryption scheme supports any polynomial time computation on ciphertext. In 2009, Gentry showed the first construction of an FHE scheme [9] that can do any number of addition and multiplication operations on encrypted data.

To explain FHE, say ciphertext *c*_*i*_ is the encrypted form of plaintext *m*_*i*_, where *m*_*i*_ and *c*_*i*_ are elements of a ring (the operations of the ring are addition and multiplication). In FHE, if a function *f* consists of addition and multiplication in the ring, then *decryption* (*f* (*c*_1_,*c*_2_,...,*c*_n_)) = *f* (*m*_1_,*m*_2_,...,*m*_n_). Generally, *f* is expressed by an arithmetic circuit over Gallois field(2). This is equivalent to a Boolean circuit with exclusive OR and AND gates.

In the existing FHE schemes, a certain amount of noise needs to be introduced in the ciphertexts to ensure data confidentiality. This noise grows while performing homomorphic operations on ciphertexts. In particular, a homomorphic multiplication operation increases the size of the ciphertext abruptly. For instance, if 2 input ciphertexts have size *M* and *N*, then the output ciphertext will be of size *M*+*N*−1. If the amount of noise becomes too high, then the ciphertext cannot be decrypted correctly. To perform any number of homomorphic operations, the noise of the ciphertexts needs to be reduced. As mentioned before, this can be done using a method known as *bootstrapping* [9], which is computationally expensive.

In use cases where only a predetermined number of computational operations needs to be done, the costly bootstrapping process can be avoided by using an SWHE scheme [23]. This scheme is often more efficient than using an FHE scheme with bootstrapping. SWHE schemes use a method called *relinearization* [15,24] to reduce the size of the ciphertext.

**Table 1:** Partial list of homomorphic encryption schemes.

Cryptosystem	Homomorphism
Goldwasser and Micali [19], Paillier [20]	Additive
Rivest et al [21], ElGamal [22]	Multiplicative
Boneh et al [18]	Both

#### Intel Software Guard Extensions

Intel SGX is a collection of extensions to the Intel architecture that mostly concentrates on the issue of running applications on a remote machine managed by an untrusted party. SGX enables parts of an application to run within secure portions of the central processing unit called *enclaves*. Untrusted entities, including system software, cannot access the enclave. SGX guarantees that the code and information inside an enclave cannot be manipulated from outside the enclave. Two SGX features facilitate provisioning of sensitive data to an enclave: attestation and sealing.

SGX enclaves are generated without privacy-sensitive information. Privacy-sensitive information is provisioned after the enclave has been appropriately instantiated. This process of demonstrating that an application has been correctly instantiated within an enclave is called *attestation* [25].

At the point when an enclave is instantiated, SGX protects its data until they are kept within the enclave. In any case, when the enclave procedure terminates, the enclave will be destroyed and all related data will be lost. So, for later use, data should be stored outside the enclave. *Sealing* is the procedure that is used to store encrypted data to ensure that only the same enclave would be capable of unsealing them back to their previous form.

### System Architecture

**Figure 1:**
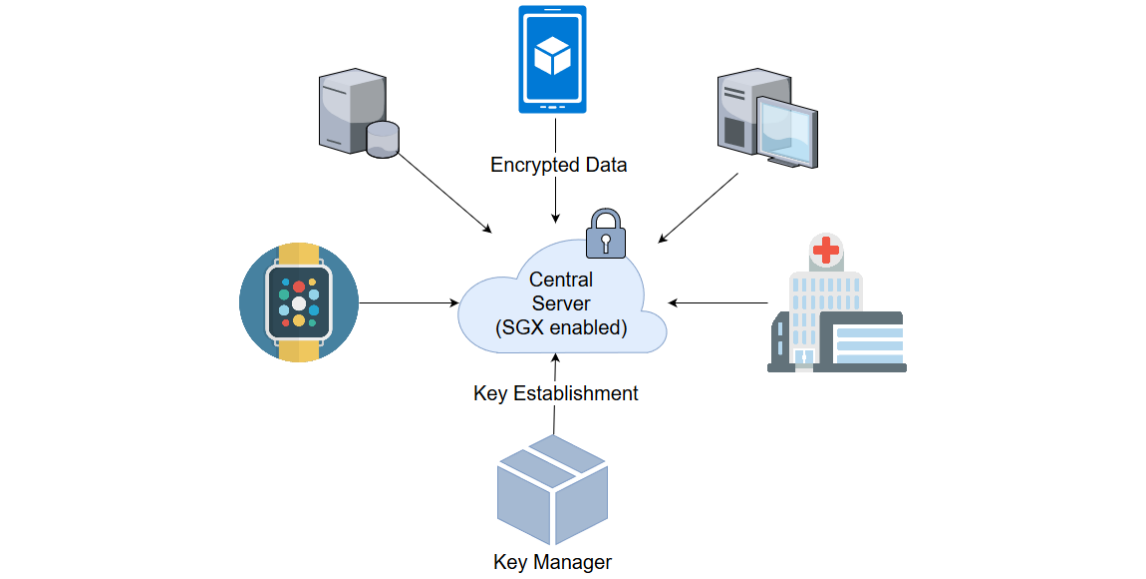
Block diagram of the system architecture. SGX: Software Guard Extensions.

Our proposed framework has three main entities (Figure 1).

#### Data Owners

These parties are geographically distributed and possess databases. Data can come from a variety of sources, including cell phones, wearable sensors, and relational databases. Data owners send encrypted intermediary results to the central server so that it can analyze the combined dataset.

#### Key Manager

This generates and distributes the cryptographic keys that will be used for data encryption and decryption in different stages of our proposed framework. Each data owner gets a public key from the key manager and uses it for encrypting data.

#### Central Server

The central server maintains communication with all the other entities of the framework. It receives data from the data owners and computes the final result using SWHE and secure hardware.

### Threat Model

In proposing this framework, our goal was to guarantee the confidentiality of data provided by different data owners. We assume that the central server is a semihonest party (also referred to as honest-but-curious), where it obeys the system protocol but may try to infer sensitive information by analyzing the system logs or received information [26].

We assume that the computation runs in an SGX-enabled central server. SGX architecture enables the central server to perform any computation securely on data provided by different data owners. We assume that the processor of the central server works properly and is not compromised. We trust the design and implementation of SGX and all cryptographic operations performed by it.

In general, side-channel attacks against SGX can be classified into two categories: physical attacks (where the attacker has physical access to the machine) and software attacks (these are launched by any malicious software running in the same machine) [27]. There has been no known successful physical attack against SGX. However, it is possible to exploit a type of software attack known as a *synchronization bug* [28]. Synchronization bugs are possible to exploit because an untrusted operating system can manipulate the thread scheduling of enclaves. However, it is only applicable for multithreaded applications, whereas our application is single threaded.

There is another type of well-known software attack, which is called a *page-fault attack* [16]. As the page tables are maintained in the operating system kernel and operated by the untrusted system software, page table entries can be manipulated to attack enclaves. But, since enclave pages are permission protected, malicious system software cannot compromise their integrity by manipulating them. However, Xu et al [16] showed that, by clearing the present flag in the corresponding page table entries, the malicious software can generate traces of page access from the enclave. Although an adversary can observe access to different enclave pages, enclave memory can be treated as private at page-level granularity (4 kB) [29]. In other words, a different access to an enclave page is indistinguishable to an adversary. Further research is required to better understand the gap between the potential vulnerabilities of SGX and proposed defense mechanisms. Most of the existing defense mechanism have been developed to address the page-fault side-channel attacks [29-31]. However, these mechanisms may not be effective for future attacks. Keeping these attacks in mind, we developed our framework to protect institutional privacy by combining the local inputs of participating institutions without decrypting them, therefore providing a higher layer of protection without introducing too much computational overhead.

We did not consider the aspects of adversarial machine learning through obtained outputs. Adversarial parties may try to infer sensitive attributes of data by model inversion attacks [32,33].

### Linear Regression

Suppose we are given a set of paired observations (*x*_i_, *y*_i_) for *i*=1,2,...,*n*, and we want to generate the best-fit straight line for these points. This straight line is given by *y*=β_1_+β_2_*x*, for some β_1_,β_2_. The purpose is to explain the correlation between variable *y* and *x*. To evaluate the fit, the value of *y* is predicted that is associated with a given value of *x*. In the literature, *y* is called *the variable to be explained* (or the *dependent* variable) and *x* is called the *explanatory variable* (the *regressor*, the *covariate*, or the *independent* variable) [34] (pg 79). Consider the following simple linear regression model: *y*=β_1_+β_2_*x* +ε. Here, ε is the error we make in predicting *y*. For *i*=1,...,*n*, we obtain *n* equations: *y*_1_=β_1_+β_2_*x*_1_+ε_1_, *y*_2_=β_1_+β_2_*x*_2_+ε_2_, and *y*_n_=β_1_+β_2_*x*_n_+ε_n_.

**Figure 2:**
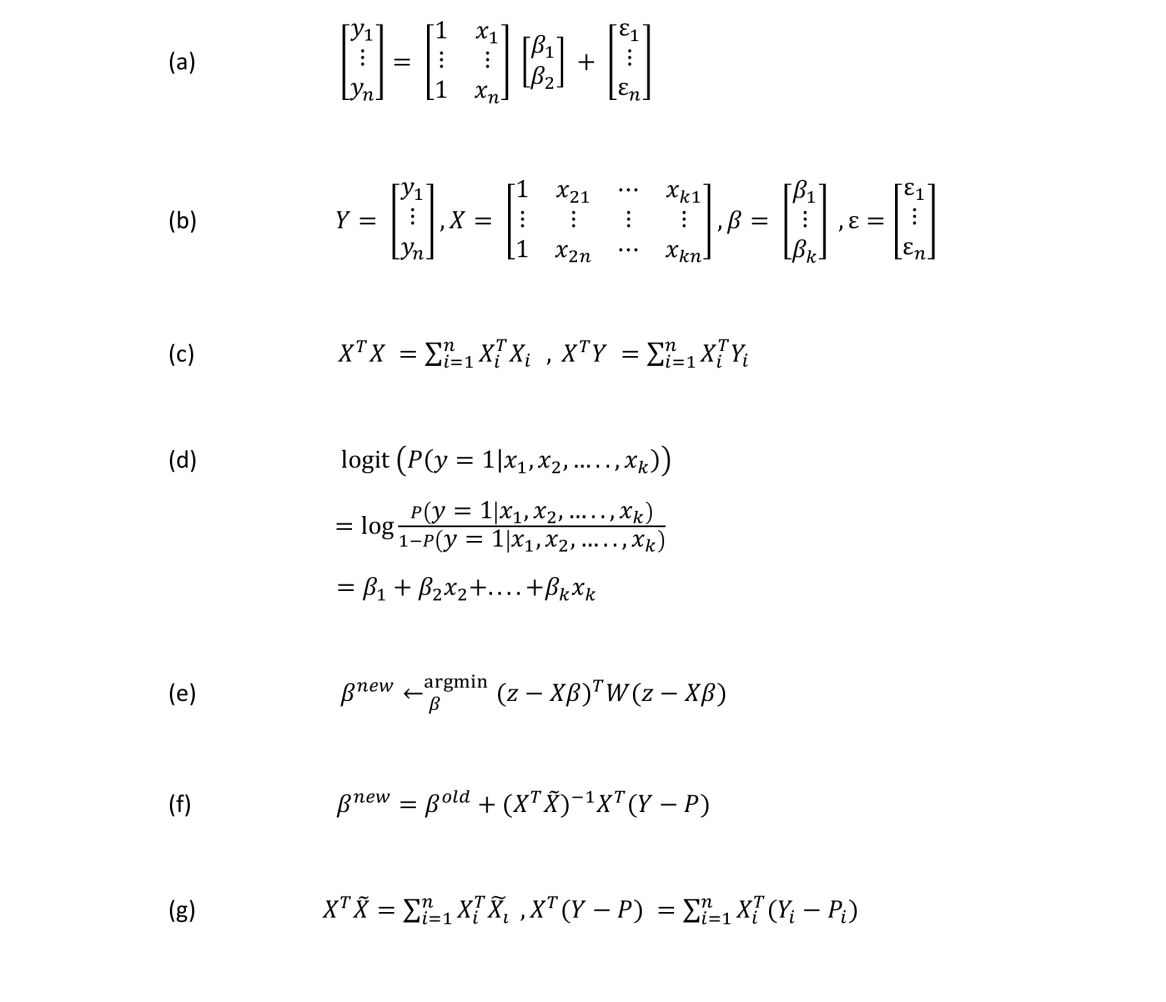
Equations used in developing the framework.

We can formulate this regression model using the matrix in Figure 2 (a).

In this way, the simple linear regression function can be represented by a short and simple equation:

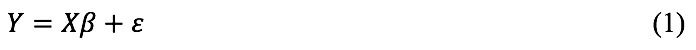



The linear regression model with several explanatory variables is known as *multiple linear regression*. This is given by

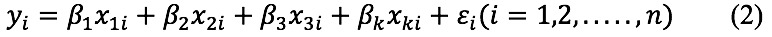



Here, *x*_1*i*_=1, for *i*=1,...,*n*. The function of Equation 2 can also be expressed in matrix form, which is more convenient, as in Figure 2 (b).

It is noteworthy that Equation 1 is also applicable for multiple linear regression.

Using the ordinary least squares estimate technique we can show that β=(*X*^*T*^*X*)^–1^*X*^*T*^*Y* (for details, see Heij et al [34], pg 79).

For secure linear regression over distributed data, each data owner *D*_*i*_ computes *X*^*T*^_*i*_*X*_*i*_ and *X*^*T*^_*i*_*Y*_*i*_ locally on plaintext. *D*_*i*_ then encrypts *X*^T^_i_*X*_i_ and *X*^*T*^_*i*_*Y*_*i*_ using homomorphic encryption. After receiving these intermediary results from all of the data owners, the central server then adds these using homomorphic addition operations to construct *X*^*T*^*Y* and *X*^*T*^*X* (equation from Figure 2 [c]). Further computation is performed inside the enclave after decryption. Textbox 1 shows our secure linear regression algorithm.

**Figure 3:**
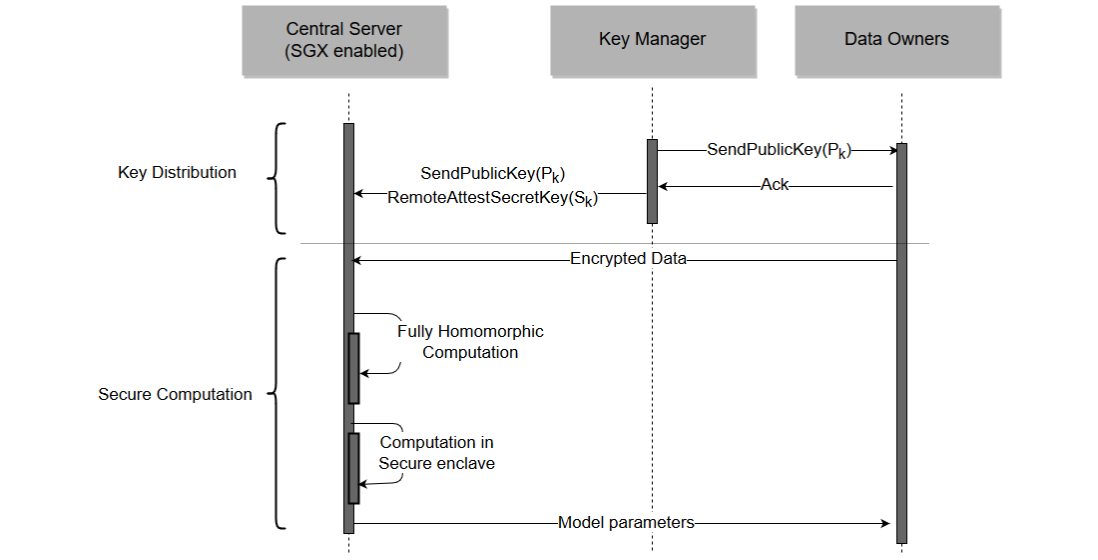
Sequence diagram of our proposed framework. Ack: acknowledge; SGX: Software Guard Extensions.

Figure 3 illustrates the sequence diagram of our proposed method. At first, the key manager establishes the public key and the private key. The private key is sent to the central server securely using remote attestation. The data owners then encrypt their data with the public key and send the encrypted data to the central server. Finally, the central server computes the model parameters.

**Algorithm 1: secure linear regression.**

**Input**: Each data owner *D*_*i*_ provides encrypted *X*^*T*^_*i*_*X*_*i*_ and *X*^*T*^_*i*_*Y*_*i*_.

**Output**: Model parameters (β)

1. Perform homomorphic addition over *X*^*T*^_*i*_*X*_*i*_ for each data owner *i*.
2. Perform homomorphic addition over *X*^*T*^_*i*_*Y*_*i*_ for each data owner *i*.
3. Send *X*^*T*^*Y* and *X*^*T*^*X* to enclave.
4. Inside enclave, decrypt encrypted *X*^*T*^*Y* and *X*^*T*^*X*.
5. Inside enclave, compute (*X*^*T*^*X*)^–1^.
6. Finally, compute β inside enclave.

### Logistic Regression

Logistic regression extends the principles of multiple linear regression to the case where the dependent variable *y* is binary (either 0 or 1). Like in multiple linear regression, the independent variables can be categorical or continuous.

Instead of modeling the dependent variable directly, logistic regression models the probability of the dependent variable. Logistic regression uses the equation of linear regression equation (2). But, in that equation, the value of the dependent variable can fall outside [0, 1]. Therefore, a nonlinear transformation is used, which is called *logit transformation*. The logit function takes any value *x* and maps it onto a value between 0 and 1. Logit function is given by logit(*x*)=log[*p* /(1–*p*)] as in Figure 2 (d). Therefore, *probability*=(*y*=1| *x*_1_, *x*_2_,...,*x*_k_) = [exp(β_1_+β_2_*x*_2_+...+β_*k*_*x*_*k*_)]/[1+exp(β_1_+β_2_*x*_2_+...+β_*k*_*x*_*k*_)] where β_1_, β_2_,...,B_*k*_ are unknown constants analogous to the multiple linear regression model. *Probability*=(*y*=1| *x*_1_, *x*_2_,...,*x*_*k*_) denotes the probability that input (*x*_1_, *x*_2_,...,*x*_*k*_) belongs to default class (*y*=1).

Logistic regression models are generally fit by maximum likelihood by using the conditional probability of *y* given *x*. Here, the Newton-Raphson method is used to solve the coefficients.

Let *X* represent the matrix of *x*_*i*_ values, *Y* represent the vector of *y*_*i*_ values, *P* be the vector of fitted probabilities with the *i*th element *p* (*x*_*i*_;β^*old*^), and *W* be an *n* × *n* diagonal matrix of weights with *i*th diagonal element *p* (*x*_*i*_;β^*old*^)(1– *p* [*x*_*i*_;β^*old*^]). Then a Newton step is as follows:

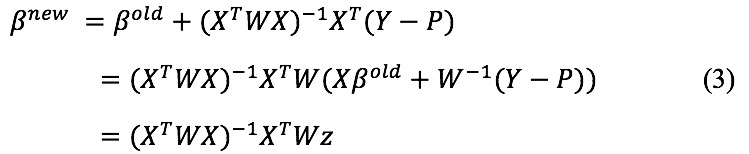



In the second and third steps, the Newton step is expressed as a weighted least squares step, with the response *z*= *X* β^*old*^+*W*^-1^(*Y*–*P*). This method is also known as iteratively reweighted least squares, since each iteration solves the weighted least squares problem (see Friedman et al [35] for details), as in Figure 2 (e).

In practice, the *W* matrix is not computed explicitly because its size could be huge. If we have 1000 rows of training data, matrix *W* would have 1,000,000 cells. For this reason, direct matrix operations with *W* may be very inefficient. Notice the beta update equation (Equation 3) has a term, *WX*, which means the matrix product of *W* and *X*. Because most of the values in *W* are zero, most of the matrix multiplication terms are also zero. This allows *W* times *X* to be computed directly from *P* and *X*, without explicitly constructing *W*. Several of the mathematical references that describe iteratively reweighted least squares with the Newton-Raphson algorithm for logistic regression use the symbol [*X* tilde] for the product of *W* and *X*. It is generally written as in Figure 2 (f).

For secure logistic regression over distributed data, each data owner *D*_*i*_ computes *X*^*T*^_*i*_[*X* tilde]_*i*_ and *X*^*T*^_*i*_(*Y*_*i*_– *P*_*i*_) locally on plaintext. *D*_*i*_ then encrypts *X*^*T*^_*i*_[*X* tilde]_*i*_ and *X*^*T*^_*i*_(*Y*_*i*_– *P*_*i*_) using homomorphic encryption. After receiving these intermediary results from all the data owners, the central server then adds these using homomorphic addition operations to construct *X*^*T*^[*X* tilde] and *X*^*T*^(*Y*– *P*) (equation from Figure 2 [g]). Further computation is performed inside the enclave after decryption. After computing β, the central server sends β to all of the data owners. For the next iteration, data owner *i* computes *X*^*T*^_*i*_[*X* tilde]_*i*_ and *X*^*T*^_*i*_(*Y*_*i*_– *P*_i_) using new β (received from the central server) and sends these intermediary results to the central server. The central server then updates β using newly received *X*^*T*^_*i*_[*X* tilde]_*i*_ and *X*^*T*^_*i*_(*Y*_*i*_– *P*_*i*_). In this way, iterations continue until parameters converge. Textbox 2 shows our secure logistic regression algorithm.

### Implementation

We developed our proposed framework using C++. For SWHE, we used the Simple Encrypted Arithmetic Library (SEAL) [24]. SEAL is an easy-to-use homomorphic encryption library, with no external dependencies. There is another homomorphic encryption framework called HElib [36], but we chose to use SEAL for its simplicity.

**Algorithm 2: secure logistic regression.**

**Input:** Each data owner *D*_*i*_ provides encrypted *X*^*T*^_*i*_[*X* tilde]_*i*_ and *X*^*T*^_*i*_(*Y*_*i*_– *P*_*i*_), and β is initialized to an all-zero vector.

**Output:** Model parameters

1. Receive encrypted *X*^*T*^_*i*_[*X* tilde]_*i*_ and *X*^*T*^_*i*_(*Y*_*i*_– *P*_*i*_) from each data owner *D*_*i*_.
2. Perform homomorphic addition over *X*^*T*^_*i*_[*X* tilde]_*i*_ for each data owner *D*_*i*_.
3. Perform homomorphic addition over *X*^*T*^_*i*_(*Y*_*i*_– *P*_*i*_) for each data owner *D*_*i*_.
4. Send encrypted *X*^*T*^[*X* tilde] and *X*^*T*^(*Y*– *P*) to enclave.
5. Inside enclave, decrypt *X*^*T*^[*X* tilde] and *X*^*T*^(*Y*– *P*).
6. Update β^new^=β^old^+(*X*^*T*^[*X* tilde])^–1^*X*^*T*^(*Y*– *P*).
7. If the stopping criteria are satisfied, then stop; otherwise, send β to each data owner and go to step 1.

**Table 2:** Parameters used for the Simple Encrypted Arithmetic Library.

Parameters	Value
Polynomial modulus	*x*^1024^+1
Plaintext modulus	1<<8
Decomposition bit count	32
No. of coefficients reserved for fractional part	64

**Table 3:** Size of datasets used for experiments.

Records	Dataset	
	Haberman	Low Birth Weight Study
No. of instances	270	488
No. of features	3	8

### Experimental Settings and Dataset

We performed experiments in a machine with an Intel Core i7-6700 (3.40 GHz) processor and 8 GB memory (Intel Corporation, Santa Clara, CA, USA). We used Intel SGX software development kit version 1.7. We simulated 2 data owners and the central server in this machine. Table 2 shows the SEAL parameters.

We performed experiments using Haberman’s survival dataset from the University of California, Irvine, Machine Learning Repository [37] and the Longitudinal Low Birth Weight Study dataset from Hosmer and Lemeshow [38]. The records of the datasets were evenly distributed between the 2 data owners.

Table 3 lists the datasets we used with their sizes.

## Results

Table 4 shows the experimental results. For SWHE, most of the computation time was due to homomorphic operations. Our proposed framework avoided expensive homomorphic multiplication by transferring the later phase of computation to the secure hardware. In addition, we needed to decrypt only the intermediary results, not every individual attribute value. Consequently, our proposed framework was more efficient than the solely secure hardware (SWHE)-based technique (where every individual attribute needs to be decrypted) and the SWHE-based technique (which involves many expensive homomorphic multiplication and relinearization operations). Table 4 does not report the results for the SWHE-based technique. However, according to our empirical results, it took more than 2 hours for the Haberman dataset and more than 17 hours for the Low Birth Weight Study dataset for both kinds of regression analyses.

**Table 4:** Experimental results for computation time.

Regression analyses		Dataset	
		Haberman	Low Birth Weight Study
**Linear regression**			
	Plaintext (ms)	6	25
	Proposed method (s)	8.991	39.382
	Secure hardware (SWHE^a^) (s)	259.908	880.228
	Secure hardware (AES^b^) (s)	4.30	8.54
**Logistic regression**			
	Plaintext (ms)	171	886
	Proposed method (s)	27.037	162.544
	Secure hardware (SWHE) (s)	264.669	904.718
	Secure hardware (AES) (s)	4.65	8.64

^a^SWHE: somewhat homomorphic encryption.

^b^AES: Advanced Encryption Standard.

**Table 5:** Storage overhead for the secure hardware approach.

Overhead before and after encryption	Dataset	
	Haberman	Low Birth Weight Study
Before encryption (kB)	3.8	28
After encryption (SWHE^a^) (MB)	30.3	123
After encryption (AES^b^) (kB)	36	143

^a^SWHE: somewhat homomorphic encryption.

^b^AES: Advanced Encryption Standard.

We want to emphasize that, although the secure hardware (Advanced Encryption Standard [AES]) method is faster, state-of-the-art attack models targeting SGX show that solely secure hardware-based approaches might expose data from participating institutions to potential attackers (as explained above). Our method, although a little bit slower, preserves such institutional privacy by combining the local inputs without decrypting them; therefore, it offers a stronger security guarantee without imposing too much computation or storage cost. In this way, our proposed hybrid model provides a good trade-off in terms of security and efficiency.

Table 5 shows the storage overhead of the solely secure hardware-based approach. For SWHE, times required to encrypt the datasets were 4.37 minutes for the Haberman dataset and 18.46 minutes for the Low Birth Weight Study dataset. For AES, times required to encrypt the datasets were 14 milliseconds for the Haberman dataset and 38 milliseconds for the Low Birth Weight Study dataset.

## Discussion

### Comparison With Prior Work

There is a homomorphic encryption-based implementation of linear regression [14], which required 2 days to compute on a dataset containing 51,000 input vectors of 22 features with a key size of 1024 bits. That matrix inversion procedure took 1 day to complete because matrix inversion is a very expensive computational task in homomorphic encryption. In our proposed method, we performed matrix inversion on plaintext in secure hardware, which is much more efficient.

Hall et al [14] proposed an iterative matrix inversion algorithm, which introduces approximation errors when a fixed number of iterations is used. Their method offers a low accuracy of 10^–3^. Precision can be slightly improved by choosing greater values for the 2 constants used by their method. However, this would require a larger public key, which would introduce significant computation overhead. In contrast, in our proposed method, there is no approximation error: the regression coefficients are completely identical to the plaintext results.

### Security Discussions

In the Methods (Threat Model subsection), we discussed the security of SGX, specifically different side-channel attacks on SGX, and how we treat those attacks in our proposed framework. Addressing these attacks, we developed our framework in such a way that it can protect institutional privacy by combining the local inputs of participating institutions without decrypting them. This approach provides a higher layer of security without imposing too much computational cost.

In our proposed method, only intermediate values (eg, *X*^*T*^*Y*, *X*^*T*^*X*) are decrypted inside secure hardware. Even if the hardware is compromised (or, in case of a side-channel attack), it is not possible to retrieve any sensitive attribute from those intermediary results. Hence, our proposed hybrid model not only achieves good performance but also guarantees stronger security than the solely SGX-based techniques. Dowlin et al [24] and Pass et al [25] discussed the security of SEAL and Intel SGX further.

A symmetric cryptosystem like AES requires *n* remote attestations to distribute the key to *n* data owners, which results in much more network communication, which might be prone to attack. In contrast, our proposed framework relies on public-key cryptography, where the data owners use a public key to encrypt their data published by the key manager. In this way, our proposed method reduces the attack surface of the system model, makes key distribution much simpler, and avoids additional communication overhead.

### Limitations

There are some limitations of our proposed framework.

First, we did not consider the issue of model privacy. Several works based on differential privacy have addressed inference attacks (eg, model privacy [39]). These solutions are complementary to our proposed method and can be readily incorporated into a single framework.

Second, the central server of our proposed method must be SGX enabled; that is, it must use an Intel processor of sixth generation or later.

Third, since computing coefficients for logistic regression require multiple iterations, all parties must be synchronized until coefficients converge. However, linear regression does not require multiple iterations. So, in this case, parties can be offline just after sending their intermediary results.

### Generalizability

Others have addressed training machine learning models (eg, support vector machines [40]) over distributed data [41,42]. Our proposed method can be easily applied to this kind of technique.

### Cost of Deployment

The Intel SGX feature is available in all Intel Skylake and Kaby Lake processors. The price of an Intel Skylake or Kaby Lake processor is identical to that of processors from other vendors (having similar configuration). Price ranges from US $42 to US $1207 depending on configuration [43]. Recently, Microsoft started using SGX-capable servers in their Azure confidential computing service [44]. Azure confidential computing is offering the developers the ability to develop applications on top of Intel SGX software development kit. Apparently, there will be no significant additional charge for using this service.

### Conclusion

In this age of big data, data need to be analyzed to uncover valuable insights and patterns. But this kind of analysis poses a threat to individual privacy, since data often contain sensitive information. In this paper, we address this data security and privacy issue and propose a hybrid cryptographic framework to overcome the limitations of the existing cryptographic techniques. We think that secure hardware–assisted predictive analysis of biomedical data is very promising for health care and medical research.

In future work, we will investigate the applicability of our proposed method to other learning algorithms such as neural networks, support vector machines, and decision trees.

## Appendix

Multimedia Appendix 1 Related works.

## Abbreviations

AES: Advanced Encryption Standard
FHE: fully homomorphic encryption
GLORE: grid binary logistic regression
HIPAA: Health Insurance Portability and Accountability Act
PIPEDA: Personal Information Protection and Electronic Documents Act
SEAL: Simple Encrypted Arithmetic Library
SGX: Software Guard Extensions
SWHE: somewhat homomorphic encryption
