# Supplementary material for: Secure and Efficient Regression Analysis Using a Hybrid Cryptographic Framework: Development and Evaluation
Source: JMIR Med Inform. 2018 Mar 5;6(1):e14. doi: 10.2196/medinform.8286 (PMC5859787; doi:10.2196/medinform.8286)
Supplement: Multimedia Appendix 1 [file medinform_v6i1e14_app1.pdf]

Appendix
Secure and Efficient Regression Analysis Using a Hybrid Cryptographic Framework

1 Related Works

In this section, we discuss some of the existing works that focus on regression analysis in a privacy-preserving way. Table 1 demonstrates some of the recent works in this area in chronological order. Each of the following Subsections cover a certain secure computation scheme.

1.1 Homomorphic Encryption

Homomorphic encryption [1] enables other parties to perform computation on the data without possession of the private key.

There are many applications of homomorphic encryption in privacy-preserving machine learning [2, 3, 4, 5]. Some of these target regression analysis [4, 5]. Hall et al. [5] proposed a multiple linear regression analysis technique based on homomorphic encryption. Their main idea is, since computing regression coefficients is basically done by matrix products, a secure method can be formulated by composing secure matrix products. Another work by Bos et al. [4] demonstrates a private predictive analysis (based on logistic regression) on encrypted medical data using homomorphic encryption.

Homomorphic encryption is impractical as it comes with huge computational and storage overhead. Some practical variants of homomorphic encryption scheme have been proposed over the last few years [6, 7, 8]. However, the overhead issue still persists.

Table 1: Previous research works in privacy-preserving regression analysis

Technique	Year	Regression Type	Differential Privacy	Homomorphic Encryption	Garbled Circuit	Intel SGX
Chaudhuri et al. [9]	2011	Linear	✓			
Lei et al. [10]	2011	Linear	✓			
Hall et al. [5]	2011	Linear		✓		
Zhang et al. [11]	2012	Linear, logistic	✓			
Wu et al. [12]	2012	Logistic			✓	
Valeria et al. [13]	2013	Ridge		✓	✓	
Bos et al. [4]	2014	Logistic		✓		
Wang et al. [14]	2016	Exact logistic		✓		
Shi et al. [15]	2016	Logistic			✓	
Ohrimenko et al. [16]	2016	–				✓
Our proposal	2017	Linear, logistic		✓		✓

1.2 Garbled Circuit

In the mid 80s, Yao proposed garbled circuits [17] in the context of secure two-party computation, which can compute a function f on input x without exposing anything about f or x. So, a malicious party cannot learn anything about the function f or the input x other than the result $f(x)$. It should be noted that the term *circuit* in this context means, boolean circuit.

Valeria et al. [13] implemented an evaluator for computing regression coefficient that uses linear homomorphism in the first phase to perform all the linear operations. In the second phase, it uses garbled circuit for non-linear computations since garbled circuit is much more efficient than homomorphic encryption for this

purpose.
However, there are some critical issues of garbled circuits.

1. First of all, standard garbled circuits suffer from one limitation: they offer no security if used on more than one inputs. In other words, garbled circuits are not reusable. Consequently, evaluating the circuit on a new input requires a completely new garbling of the circuit.

2. Another problem with garbled circuits is that the communication complexity is proportional to the size of the circuit. This makes garbled circuits inefficient from the communication perspective [18, Page 22]. However, with homomorphic encryption, the communication complexity is much less.
   For instance, consider a scenario, where encrypted clinical data is stored in the cloud, and a researcher executes private prediction queries on this massive clinical data set. In this case, the communication complexity of a private query is extremely high since the garbled circuit used to represent the query is proportional to the size of the dataset. On the contrary, the communication complexity of such a query in homomorphic encryption scheme is proportional to the size of the encrypted response to the query.

3. Finally, garbled circuit-based techniques need complex circuit design and optimization for each particular computation. Thus, it is not very flexible.

1.3 Differential Privacy

Solutions based on differential privacy [19] add noise to the data to preserve individual privacy.

There are also some works on differentially private regression analysis [20, 9, 10, 11]. The solution proposed by Chaudhuri et al. [20, 9] is applicable only for linear regression. Lei [10] proposed another technique where in the first step, they generate noisy histogram from the input data. Then, from the noisy histogram they generate synthetic data by preserving statistical property of the histogram. In the final step, they uses synthetic data to compute the regression results. Finally, Zhang et al. [11] proposed a solution based on functional mechanism. Instead of perturbing the results, they perturb the objective function (cost function) of the regression analysis.

Noise added by differentially private techniques reduces data utility, and makes statistical analysis very difficult. Also, differential privacy requires one trusted entity who can access the integrated dataset. In addition, in client-server architecture, where a client executes query on the database stored in the server, differential privacy is not applicable for several types of queries [21].

1.4 Secure Hardware

Intel Software Guard Extensions [22, 23] is a set of extensions to the Intel architecture, which provides support to run an application inside protected execution area of a processor. Among the state-of-the-art secure computation schemes, Intel SGX is the most efficient. For example, an SGX-based MapReduce framework [24] demonstrates an overhead of only 8% to achieve read/write integrity. This is a significant benefit of SGX in comparison to other secure computation techniques like garbled circuit and homomorphic encryption, which generally increase the computational overhead several times.

There are no secure hardware based techniques that target regression analysis (to the best of our knowledge). However, Ohrimenko et al. [16] worked on some machine learning algorithms using Intel SGX.

Although, SGX is very efficient from computation and storage point of view, the security guarantee of SGX is yet to be fully established due to some recently proposed side-channel attacks against SGX [25, 26, 27].

References

[1] C. Gentry *et al.*, "Fully homomorphic encryption using ideal lattices." in *STOC*, vol. 9, 2009, pp. 169–178.

[2] N. Dowlin, R. Gilad-Bachrach, K. Laine, K. Lauter, M. Naehrig, and J. Wernsing, "Cryptonets: Applying neural networks to encrypted data with high throughput and accuracy," in *International Conference on Machine Learning ICML*, vol. 48, 2016, pp. 201–210.

[3] T. Graepel, K. Lauter, and M. Naehrig, "Ml confidential: Machine learning on encrypted data," in *International Conference on Information Security and Cryptology*. Springer, 2012, pp. 1–21.

[4] J. W. Bos, K. Lauter, and M. Naehrig, "Private predictive analysis on encrypted medical data," *Journal of biomedical informatics*, vol. 50, pp. 234–243, 2014.

[5] R. Hall, S. E. Fienberg, and Y. Nardi, "Secure multiple linear regression based on homomorphic encryption," *Journal of Official Statistics*, vol. 27, no. 4, p. 669, 2011.

[6] Z. Brakerski and V. Vaikuntanathan, "Fully homomorphic encryption from ring-lwe and security for key dependent messages," in *Annual cryptology conference*. Springer, 2011, pp. 505–524.

[7] Z. Brakerski, C. Gentry, and V. Vaikuntanathan, "(leveled) fully homomorphic encryption without bootstrapping," *ACM Transactions on Computation Theory (TOCT)*, vol. 6, no. 3, p. 13, 2014.

[8] J. W. Bos, K. E. Lauter, J. Loftus, and M. Naehrig, "Improved security for a ring-based fully homomorphic encryption scheme." in *IMA Int. Conf.* Springer, 2013, pp. 45–64.

[9] K. Chaudhuri, C. Monteleoni, and A. D. Sarwate, "Differentially private empirical risk minimization," *Journal of Machine Learning Research*, vol. 12, no. Mar, pp. 1069–1109, 2011.

[10] J. Lei, "Differentially private m-estimators," in *Advances in Neural Information Processing Systems*, 2011, pp. 361–369.

[11] J. Zhang, Z. Zhang, X. Xiao, Y. Yang, and M. Winslett, "Functional mechanism: regression analysis under differential privacy," *Proceedings of the VLDB Endowment*, vol. 5, no. 11, pp. 1364–1375, 2012.

[12] Y. Wu, X. Jiang, J. Kim, and L. Ohno-Machado, "Grid binary logistic regression (glore): building shared models without sharing data," *Journal of the American Medical Informatics Association*, vol. 19, no. 5, pp. 758–764, 2012.

[13] V. Nikolaenko, S. Ioannidis, U. Weinsberg, M. Joye, N. Taft, and D. Boneh, "Privacy-preserving matrix factorization," in *Proceedings of the 2013 ACM SIGSAC conference on Computer & communications security*. ACM, 2013, pp. 801–812.

[14] S. Wang, Y. Zhang, W. Dai, K. Lauter, M. Kim, Y. Tang, H. Xiong, and X. Jiang, "Healer: Homomorphic computation of exact logistic regression for secure rare disease variants analysis in gwas," *Bioinformatics*, vol. 32, no. 2, pp. 211–218, 2016.

[15] H. Shi, C. Jiang, W. Dai, X. Jiang, Y. Tang, L. Ohno-Machado, and S. Wang, "Secure multi-party computation grid logistic regression (smac-glore)," *BMC Medical Informatics and Decision Making*, vol. 16, no. 3, p. 89, 2016.

[16] O. Ohrimenko, F. Schuster, C. Fournet, A. Mehta, S. Nowozin, K. Vaswani, and M. Costa, "Oblivious multi-party machine learning on trusted processors," in *USENIX Security*, 2016.

[17] A. C.-C. Yao, "Protocols for secure computations," in *FOCS*, vol. 82, 1982, pp. 160–164.

[18] C. Gentry, "A fully homomorphic encryption scheme," Ph.D. dissertation, Stanford University, 2009.

[19] C. Dwork, "Differential privacy," in *33rd International Colloquium on Automata, Languages and Programming, part II (ICALP 2006)*, vol. 4052. Venice, Italy: Springer Verlag, July 2006, pp. 1–12. [Online]. Available: https://www.microsoft.com/en-us/research/publication/differential-privacy/

[20] K. Chaudhuri and C. Monteleoni, "Privacy-preserving logistic regression," in *Advances in Neural Information Processing Systems*, 2009, pp. 289–296.

[21] A. Groce, J. Katz, and A. Yerukhimovich, "Limits of computational differential privacy in the client/server setting," in *Theory of Cryptography Conference*. Springer, 2011, pp. 417–431.

[22] M. Hoekstra, R. Lal, P. Pappachan, V. Phegade, and J. Del Cuvillo, "Using innovative instructions to create trustworthy software solutions." in *HASP@ ISCA*, 2013, p. 11.

[23] I. Anati, S. Gueron, S. Johnson, and V. Scarlata, "Innovative technology for cpu based attestation and sealing," in *Proceedings of the 2nd international workshop on hardware and architectural support for security and privacy*, vol. 13, 2013.

[24] F. Schuster, M. Costa, C. Fournet, C. Gkantsidis, M. Peinado, G. Mainar-Ruiz, and M. Russinovich, "Vc3: trustworthy data analytics in the cloud using sgx," in *2015 IEEE Symposium on Security and Privacy*. IEEE, 2015, pp. 38–54.

[25] Y. Xu, W. Cui, and M. Peinado, "Controlled-channel attacks: Deterministic side channels for untrusted operating systems," in *Security and Privacy (SP), 2015 IEEE Symposium on*. IEEE, 2015, pp. 640–656.

[26] N. Weichbrodt, A. Kurmus, P. Pietzuch, and R. Kapitza, “Asyncshock: Exploiting synchronisation bugs in intel sgx enclaves,” in *European Symposium on Research in Computer Security*. Springer, 2016, pp. 440–457.

[27] W. Wang, G. Chen, X. Pan, Y. Zhang, X. Wang, V. Bindschaedler, H. Tang, and C. A. Gunter, “Leaky cauldron on the dark land: Understanding memory side-channel hazards in sgx,” *arXiv preprint arXiv:1705.07289*, 2017.
